# Validation of Artificial Intelligence Cardiac MRI Measurements: Relationship to Heart Catheterization and Mortality Prediction

**DOI:** 10.1148/radiol.212929

**Published:** 2022-06-14

**Authors:** Samer Alabed, Faisal Alandejani, Krit Dwivedi, Kavita Karunasaagarar, Michael Sharkey, Pankaj Garg, Patrick J. H. de Koning, Attila Tóth, Yousef Shahin, Christopher Johns, Michail Mamalakis, Sarah Stott, David Capener, Steven Wood, Peter Metherall, Alexander M. K. Rothman, Robin Condliffe, Neil Hamilton, James M. Wild, Declan P. O’Regan, Haiping Lu, David G. Kiely, Rob J. van der Geest, Andrew J. Swift

**Affiliations:** From the Department of Infection, Immunity, and Cardiovascular Disease (S.A., F.A., K.D., M.A., P.G., Y.S., C.J., S.S., D.C., A.M.K.R., R.C., N.H., J.M.W., D.G.K., A.J.S.), INSIGNEO, Institute for *in silico Medicine* (S.A., J.M.W., D.G.K., A.J.S.), and Department of Computer Science (M.M., H.L.), University of Sheffield, Glossop Road, Sheffield S10 2JF, UK; Department of Clinical Radiology, Sheffield Teaching Hospitals, Sheffield, UK (S.A., K.D., K.K., M.S., Y.S., C.J., S.W., P.M.); Leiden University Medical Center, Leiden, the Netherlands (P.J.H.d.K., R.J.v.d.G.); Semmelweis University Heart and Vascular Center, Budapest, Hungary (A.T.); Sheffield Pulmonary Vascular Disease Unit, Royal Hallamshire Hospital, Sheffield, UK (R.C., D.G.K.); and MRC London Institute of Medical Sciences, Imperial College London, London, UK (D.P.O.).

## Abstract

**Background:**

Cardiac MRI measurements have diagnostic and prognostic value in the evaluation of cardiopulmonary disease. Artificial intelligence approaches to automate cardiac MRI segmentation are emerging but require clinical testing.

**Purpose:**

To develop and evaluate a deep learning tool for quantitative evaluation of cardiac MRI functional studies and assess its use for prognosis in patients suspected of having pulmonary hypertension.

**Materials and Methods:**

A retrospective multicenter and multivendor data set was used to develop a deep learning–based cardiac MRI contouring model using a cohort of patients suspected of having cardiopulmonary disease from multiple pathologic causes. Correlation with same-day right heart catheterization (RHC) and scan-rescan repeatability was assessed in prospectively recruited participants. Prognostic impact was assessed using Cox proportional hazard regression analysis of 3487 patients from the ASPIRE (Assessing the Severity of Pulmonary Hypertension In a Pulmonary Hypertension Referral Centre) registry, including a subset of 920 patients with pulmonary arterial hypertension. The generalizability of the automatic assessment was evaluated in 40 multivendor studies from 32 centers.

**Results:**

The training data set included 539 patients (mean age, 54 years ± 20 [SD]; 315 women). Automatic cardiac MRI measurements were better correlated with RHC parameters than were manual measurements, including left ventricular stroke volume (*r *= 0.72 vs 0.68; *P* = .03). Interstudy repeatability of cardiac MRI measurements was high for all automatic measurements (intraclass correlation coefficient range, 0.79–0.99) and similarly repeatable to manual measurements (all paired* t* test *P* > .05). Automated right ventricle and left ventricle cardiac MRI measurements were associated with mortality in patients suspected of having pulmonary hypertension.

**Conclusion:**

An automatic cardiac MRI measurement approach was developed and tested in a large cohort of patients, including a broad spectrum of right ventricular and left ventricular conditions, with internal and external testing. Fully automatic cardiac MRI assessment correlated strongly with invasive hemodynamics, had prognostic value, were highly repeatable, and showed excellent generalizability.

Clinical trial registration no. NCT03841344

Published under a CC BY 4.0 license.

*Online supplemental material is available for this article.*

See also the editorial by Ambale-Venkatesh and Lima in this issue.

*An earlier incorrect version appeared online. This article was corrected on June 27, 2022.*

## Graphical abstract

**Figure:**
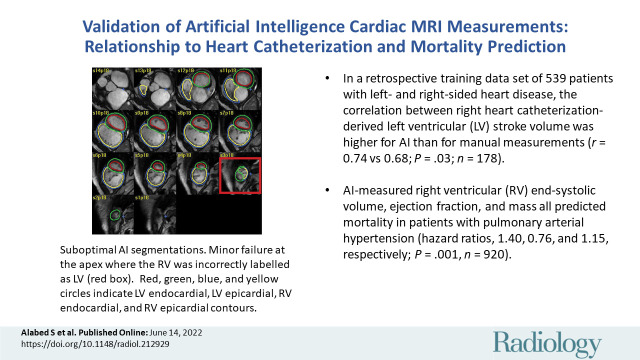

**Summary**

Artificial intelligence cardiac MRI measurements were validated and used to assess future patient mortality.

**Key Results**

■ A retrospective training data set of 539 patients with left and right heart disease was used to train an artificial intelligence (AI) model for cardiac MRI measurements.
■ Same-day cardiac MRI and right heart catheterization demonstrated strong correlation that was higher with AI measurements than with manual measurements for left ventricular stroke volume (*r* = 0.74 vs 0.68; *P* = .03; *n* = 178).
■ AI-measured right ventricular end-systolic volume, ejection fraction, and mass all predicted mortality in patients with pulmonary arterial hypertension (hazard ratios, 1.40, 0.76, and 1.15, respectively; *P* = .001; *n* = 920).

## Introduction

Cardiac MRI is the reference standard for measuring cardiac chambers and has an important role in the diagnosis and prognosis of cardiovascular disease. Manual measurements are obtained by tracing the cardiac chambers in end-diastole and end-systole, a time-consuming process that requires a specialized workforce. Efforts to automate cardiac MRI measurements have evolved over recent years (1) and have achieved comparable results to manual assessments in assessing the left ventricle (LV) (2). However, greater internal and external testing and clinical benchmarks for automatic cardiac MRI quantification are required.

Automatic assessment in the right ventricle (RV) is a challenge because of the variation in the shape, thickness, and complex anatomy, particularly at the base and outflow tract (1,3). Additionally, the RV shape can undergo extreme morphologic changes in conditions such as pulmonary hypertension (4). Automating RV assessments has the potential to improve reproducibility of RV analysis. To date, artificial intelligence (AI) biventricular segmentation studies are based on small single-center and single-vendor data sets, include limited numbers of patients with conditions affecting the RV, and do not assess RV mass (1).

The aim of our study was to develop and comprehensively evaluate an automated deep learning quantitative analysis of LV and RV cardiac MRI measurements. We also sought to assess the hypothesis that an AI cardiac MRI biventricular analysis correlates with invasive hemodynamics, predicts mortality in pulmonary hypertension, and is repeatable and generalizable.

## Materials and Methods

### Study Sample

**Figure 1:**
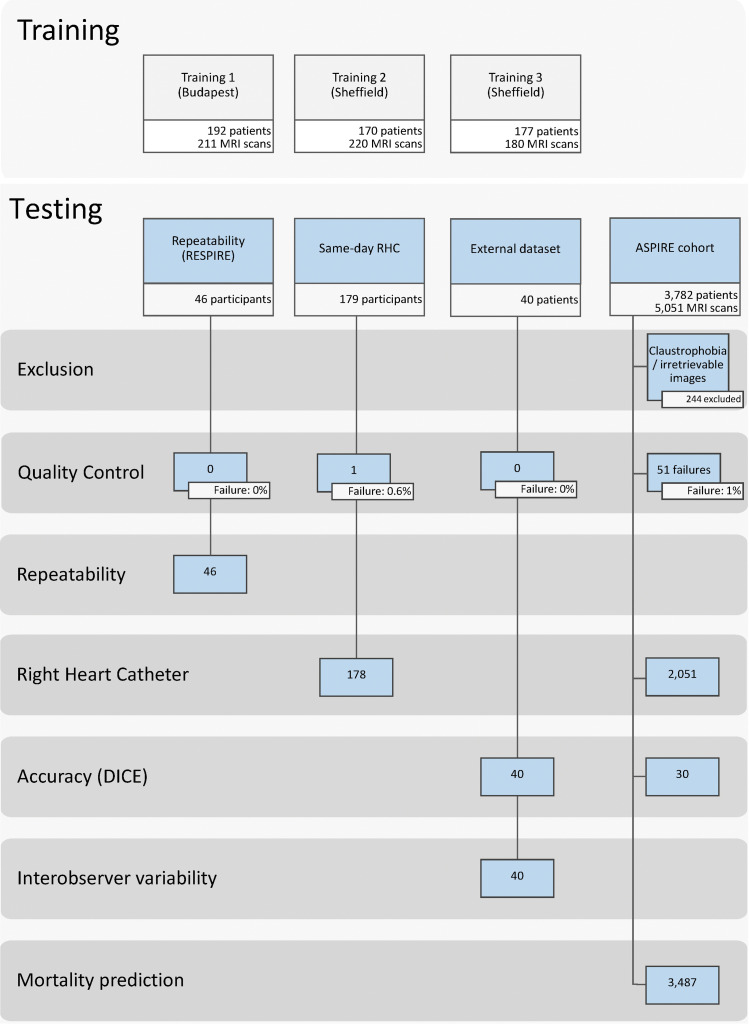
Study participant flow chart for the training and testing cohorts. ASPIRE = Assessing the Severity of Pulmonary Hypertension In a Pulmonary Hypertension Referral Centre, RESPIRE = Repeatability and Sensitivity to Change of Noninvasive End Points in Pulmonary Arterial Hypertension, RHC 5 right heart catheter.

Our study involved a retrospective training data set and a testing data set (Fig 1). The training data set included 611 studies performed at two university teaching hospitals (Sheffield Teaching Hospitals, Sheffield; and Heart and Vascular Center of Semmelweis University, Budapest) in 539 participants with various cardiac abnormalities (Appendix E1 [online]).

**Figure 2:**
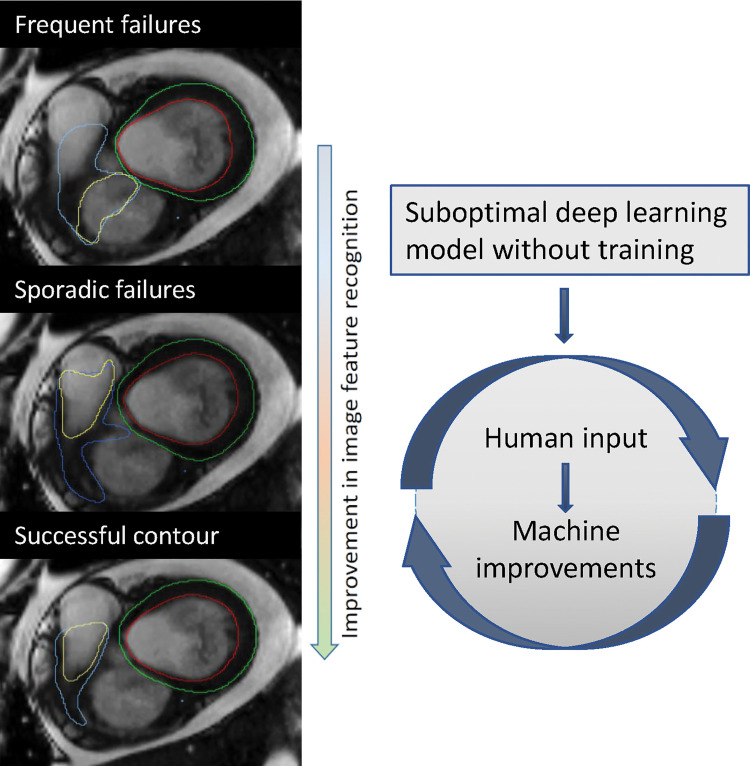
Example of improvement following additional training. This example demonstrates improvement of the right ventricular base after additional training. The first model missed the right ventricular outflow tract and included the right atrium instead (top image: yellow annotation showing right ventricular endocardial border), whereas the final model correctly included the right ventricular outflow tract and excluded the right atrium (bottom image).

The Budapest data set included 192 patients with 211 MRI studies randomly chosen in patients referred for investigation of suspected or confirmed LV disease. Overall, the data set included 180 MRI studies with LV disease and 32 normal MRI studies. The Budapest studies were performed with a Philips MRI system and was used to train the initial LV and RV segmentation model. For the Sheffield data set, consecutive patients suspected of having pulmonary hypertension who underwent cardiac MRI were identified from the Assessing the Severity of Pulmonary Hypertension In a Pulmonary Hypertension Referral Centre (known as ASPIRE) registry between 2007 and 2021 (5). Patients with incomplete, unavailable, or unretrievable short-axis stack were excluded. An off-line human-in-the-loop approach was used, wherein the initial segmentation model trained with the MRI scans acquired at Budapest was tested in the Sheffield data set and a random sample of cases that had suboptimal or failed segmentations were included for further training (Fig 2). The first round of training included 220 MRI studies and the second round included 180 studies that were again identified from suboptimal segmentations resulting from the refined segmentation model. The first, second, and final training rounds were performed with Philips, GE, and Siemens MRI systems, respectively.

For the clinical testing, four data sets were included: a prospective same-day repeatability cohort (*n* = 46); a prospective same-day right heart catheterization (RHC) cohort (*n* = 179); an external test cohort (*n* = 40) from 32 centers across England, Wales, and Scotland; and MRI studies not included in the training from the ASPIRE registry used for the assessment of mortality prediction (*n* = 3782).

Participants were recruited prospectively for our reproducibility analysis as part of the Repeatability and Sensitivity to Change of Noninvasive End Points in Pulmonary Arterial Hypertension, or RESPIRE, study (6) (ClinicalTrials.gov identifier: NCT03841344). Ethical approval for the study was granted by the local ethics committee and institutional review board (ASPIRE, reference c06/Q2308/8; REC 17/YH/0016; and RESPIRE, REC 15/YH/0269). All prospectively recruited participants gave written informed consent. All data were strictly anonymized before analysis. We followed the Checklist for Artificial Intelligence in Medical Imaging (known as CLAIM) for reporting AI studies (7).

### Imaging Procedures

***MRI protocol.—***Cardiac MRI was performed with 1.5-T MRI systems from three vendors (Signa HDx, GE Healthcare; Avanto, Siemens Solutions; and Achieva, Philips Healthcare). Multisection short-axis cine images were obtained by using a standard cardiac-gated balanced steady-state free precession sequence of 8-mm section thickness and 20 phases per cardiac cycle (Signa HDx; GE Healthcare), 6-mm section thickness and 25 phases per cardiac cycle (Avanto; Siemens Solutions), and 8-mm section thickness and 25 phases per cycle (Achieva; Philips Healthcare). The parameters (repetition time msec/echo time msec) were 3.7/1.6 (Signa HDx; GE Healthcare), 38.92/1.13 (Avanto; Siemens Solutions), and 2.72/1.36 (Achieva; Philips Healthcare). Two-dimensional phase-contrast sequences were acquired perpendicular to the long axis of the aortic lumen by using through-plane velocity encoding. All phase-contrast sequences were performed with GE MRI systems with the following imaging parameters: 5.6/2.7; section thickness, 10 mm; 20 phases; and velocity encoding, 150 cm per second in the section direction.

***Image analysis.—***Manual segmentations of biventricular epicardial and endocardial contours on short-axis stack images for the training and testing data sets were performed by seven observers (A.T., D.C., K.K., A.J.S., S.S., F.A.A., and S.A., with 19, 17, 13, 11, 4, 3, and 3 years of specialist cardiac MRI experience, respectively). All manual contours were reviewed by one author (A.J.S., a level 3 accredited cardiac MRI radiologist). Trabeculations were included in the blood pool, and the outflow tract was included for both the RV and LV. In the ASPIRE cohort, trabeculations were excluded from the blood pool (performed by D.C.). Manual segmentation for the Dice accuracy analysis was performed independently (by K.K. and A.J.S.), and for the external cohort testing (by S.A. and A.J.S). The scan-rescan segmentations were performed by a senior cardiac MRI radiographer with 3 years of experience who was not involved in the model training. All manual contouring was performed blinded to the clinical data and RHC results. Software was used for manual contouring (MASS, research version 2020; Leiden University Medical Center). A visual quality review for all segmentation was performed together by two authors (S.A. and A.J.S.) to identify the failure rate of the final segmentation model. Failed segmentations were those that resulted in visually unacceptable contours and would lead to incorrect measurement. Contours with minimal errors that were deemed to not effect cardiac MRI measurements were labeled suboptimal segmentations.

### AI Model Development

The convolutional neural network used for the experiments had a UNET-like architecture (similar in implementation to *https://github.com/dmolony3/ResUNet*) with 16 convolutional layers, including residual learning units, and was implemented by using Python (version 3.6.9; Python Software Foundation) and TensorFlow (version 1.12) (8). Input images were resampled to a fixed pixel spacing of 1 mm and cropped to a 256 × 256 matrix size and zero-filled when required. For training, the Adam optimizer method was used, the learning rate was selected as 0.001, and cross-entropy was used as the loss function. Data augmentation was performed by creating new training samples by randomly rotating, flipping, shifting, and modifying the image intensities of the original images. Each training batch included a random selection of 20 images. The fixed number of epochs was set at 30, with all images used once during every epoch. Further details on the AI model development are in Appendix E2 (online).

### Statistical Analysis

Continuous variables are presented as proportions, means ± SDs, or medians with interquartile ranges for data with asymmetric distributions. Variable standardization was performed to allow comparison of the different continuous variables on the same scale by subtracting the mean for each variable and dividing it by its SD. Cardiac MRI volumetric measurements were indexed for body surface area. Measurements were corrected for age and sex by calculating the percentage predicted values per published reference data (9,10).

The interstudy repeatability was assessed with interclass correlation coefficient (ICC) and Bland-Altman analysis to compare the scan-rescan variation in the automated and manual cardiac MRI measurements. The paired *t* test was calculated to compare the differences in scan-rescan measurements between AI and manual assessment. Spearman correlation coefficient was used to compare the LV stroke volume to the stroke volume derived from RHC and the aortic forward flow volume at the LV outflow tract measured by phase-contrast imaging. RHC stroke volume was derived by dividing cardiac output by heart rate. RV ejection fraction and ventricular mass index was correlated to RHC pulmonary vascular resistance and mean pulmonary artery pressure. Ventricular mass index was calculated as the RV end-diastolic mass–to–LV end-diastolic mass ratio (RV mass–to–LV mass ratio). The *z *test using the method by Steiger (11) was performed to test for differences between manual and AI correlations with RHC and phase-contrast imaging.

Uni- and multivariable Cox proportional hazard regression hazard ratios were calculated for both the age- and sex-adjusted cardiac MRI parameters. Collinearity was tested by using Spearman correlation test. A correlation of *r* greater than 0.8 was considered to be closely related. All patients were followed up until the all-cause mortality or administrative censoring date (June 20, 2021). No patient was lost to follow-up. The interstudy repeatability was assessed with ICC and Bland-Altman analysis to compare the scan-rescan variation in the automated and manual cardiac MRI measurements. The paired *t* test was calculated to compare the differences in the scan-rescan measurements between AI and manual assessment. The agreement between the AI and manual cardiac MRI measurements in the external test data set was analyzed with ICC and Bland-Altman plots. The accuracy of the AI contours relative to the manual contours was estimated by calculating the Dice similarity coefficient in 30 studies from an internal test data set randomly chosen from the cohort and in the 40 studies from the external test data set. The Dice score measured the ratio of overlap and distance between the manual and automatically segmented areas; a higher value indicated better accuracy of the contouring model relative to the manual segmentation. Statistical analyses were performed by using the Pingouin (version 0.5) (12) and Lifelines (version 0.26) (13) Python libraries, and graphs were produced by using the Matplotlib library (version 3.5) (14). A *P* value of .05 or less indicated statistical significance. All tests were performed at .05 level.

## Results

### Study Sample Characteristics

The total study sample included 4289 patients and 5630 cardiac MRI studies, after excluding 244 patients because of either incomplete or unretrievable imaging (Fig 1). The median age in the training data set was 58 years (IQR, 34 years), 66 years (IQR, 21 years) in the ASPIRE cohort, 67 years (IQR, 19 years) in the patients with same-day RHC, and 48 years (IQR, 22 years) in the scan-rescan patients. The ratios of women were as follows: training data set, 315 of 539 (58%); ASPIRE cohort, 2158 of 3487 (61%); patients with same-day RHC, 131 of 178 (56%); and scan-rescan patients, 35 of 46 (78%) (Table 1, 2).

**Table 1:**
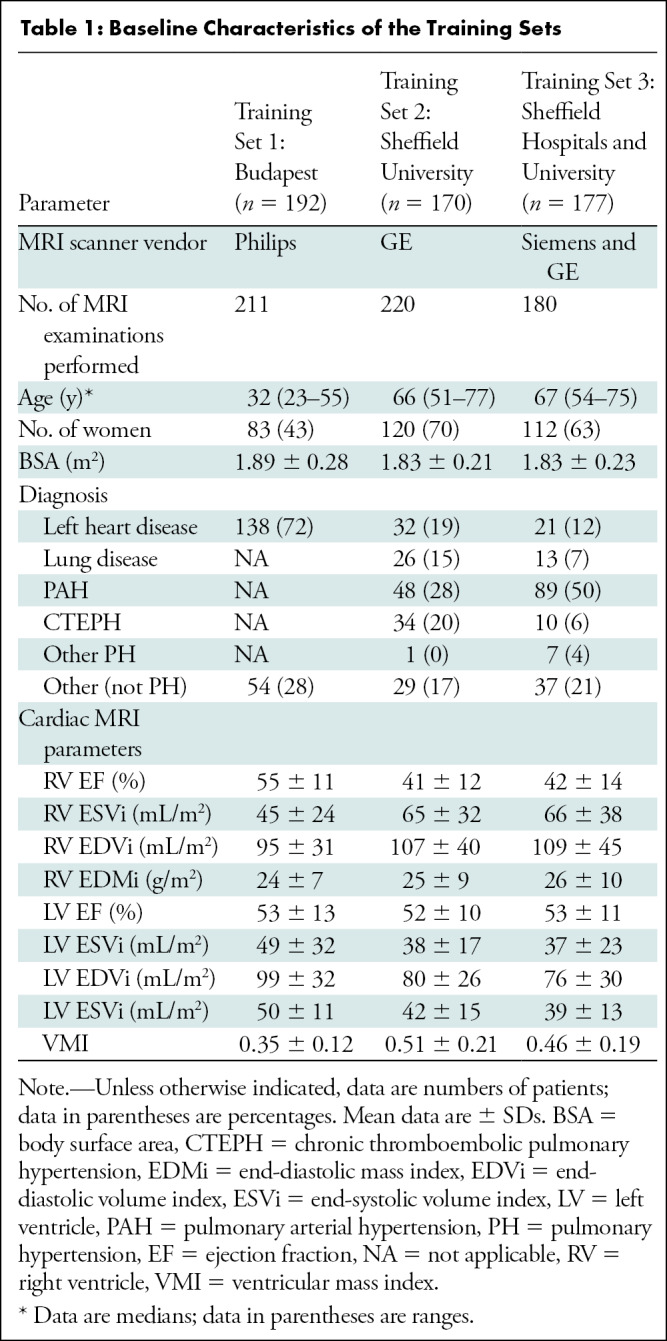
Baseline Characteristics of the Training Sets

**Table 2:**
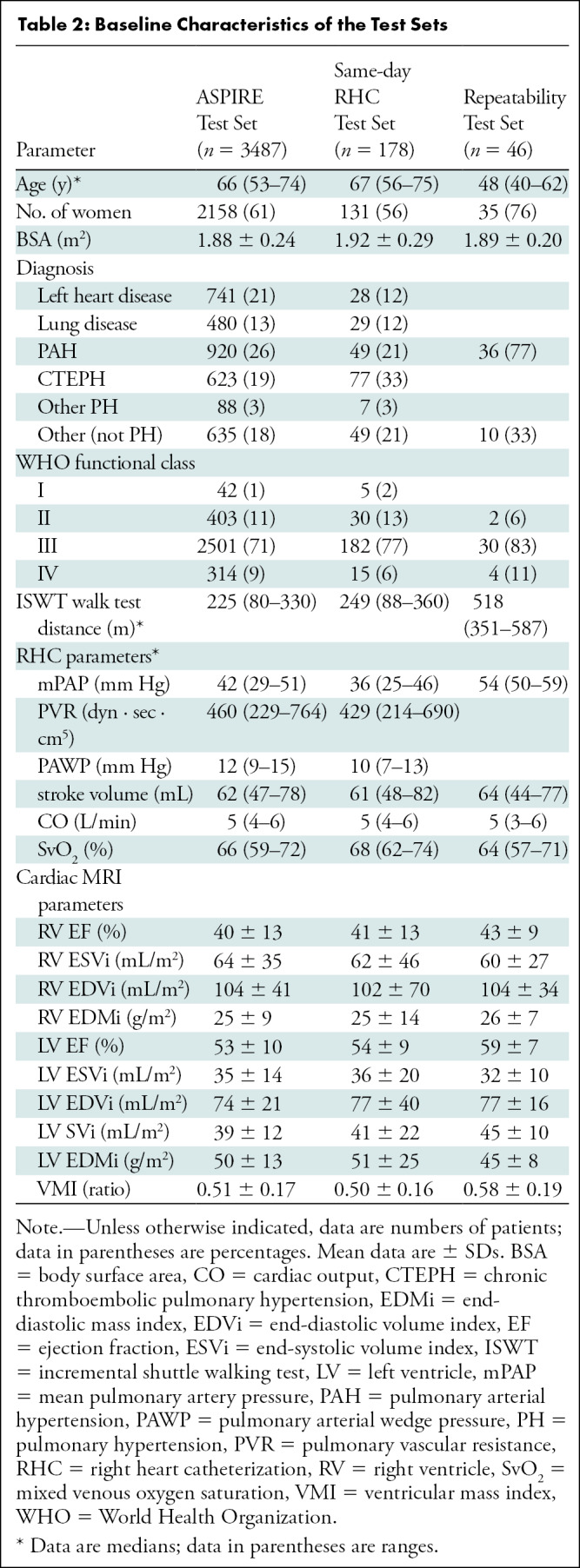
Baseline Characteristics of the Test Sets

### Quality Control

**Figure 3:**
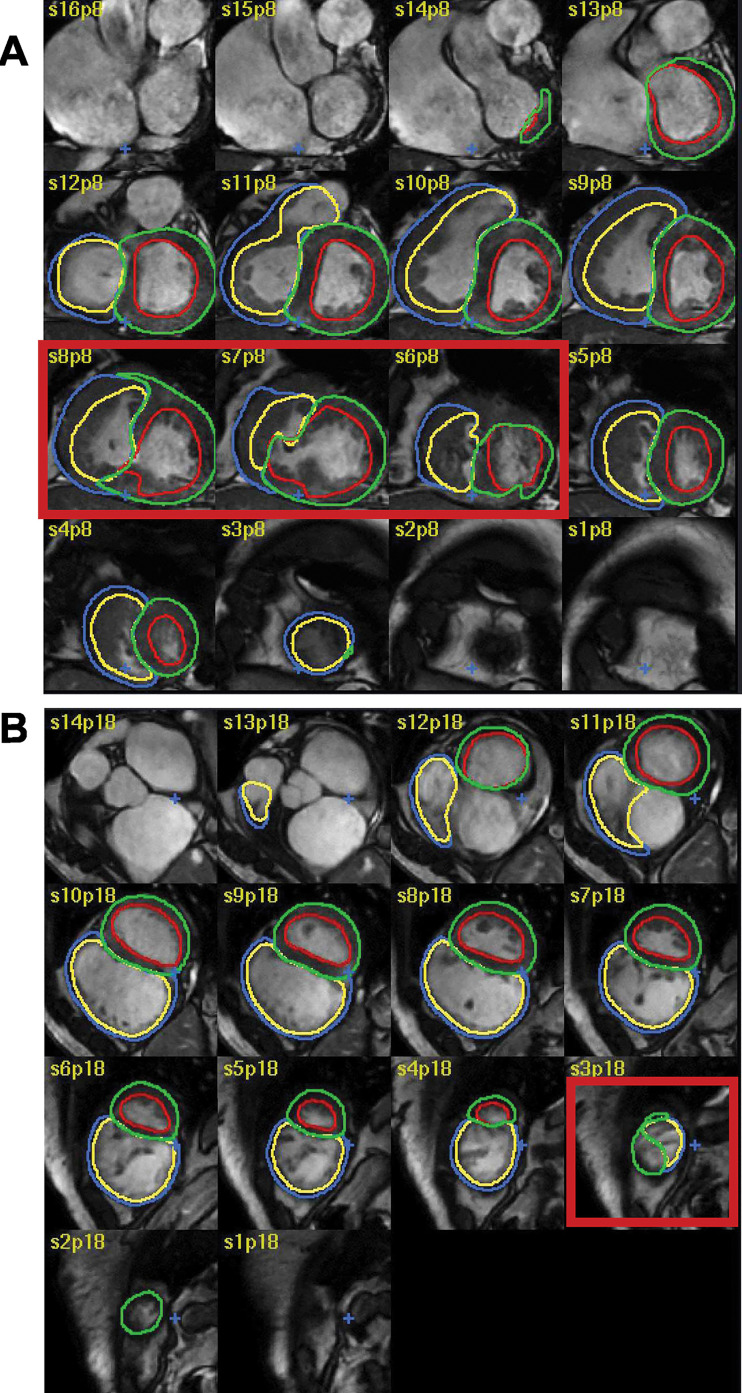
Examples of failed and suboptimal artificial intelligence (AI) segmentations. **(A)** Major failure because of congenital heart disease causing the left ventricular (LV) contours to extend into the right ventricle (RV; red box). **(B)** Minor failure at the apex where the RV was incorrectly labelled as LV (red box). The red, green, blue, and yellow circles indicate the LV endocardial, LV epicardial, RV endocardial, and RV epicardial contors, respectively.

An example of the AI segmentation of the short-axis stack throughout the cardiac cycle is shown in Movie 1 (online). The overall failure rate of the automatic segmentation was 1.0% (53 of 5316), almost exclusively caused by congenital heart diseases such as a ventricular septal defect (Fig 3A) or artifacts and technical issues affecting image quality. In 91 of 5316 studies (1.7%), there were segmentation errors mainly affecting the heart apex (Fig 3B).

**Movie 1:** Example of an automatically segmented cardiac MRI with severe RV dysfunction and RV dilatation. The AI model was particularly accurate at segmenting the RV base, which is the area typically most challenging for manual assessors.

### Correlations with Invasive Hemodynamics and Phase Contrast Flow

**Figure 4:**
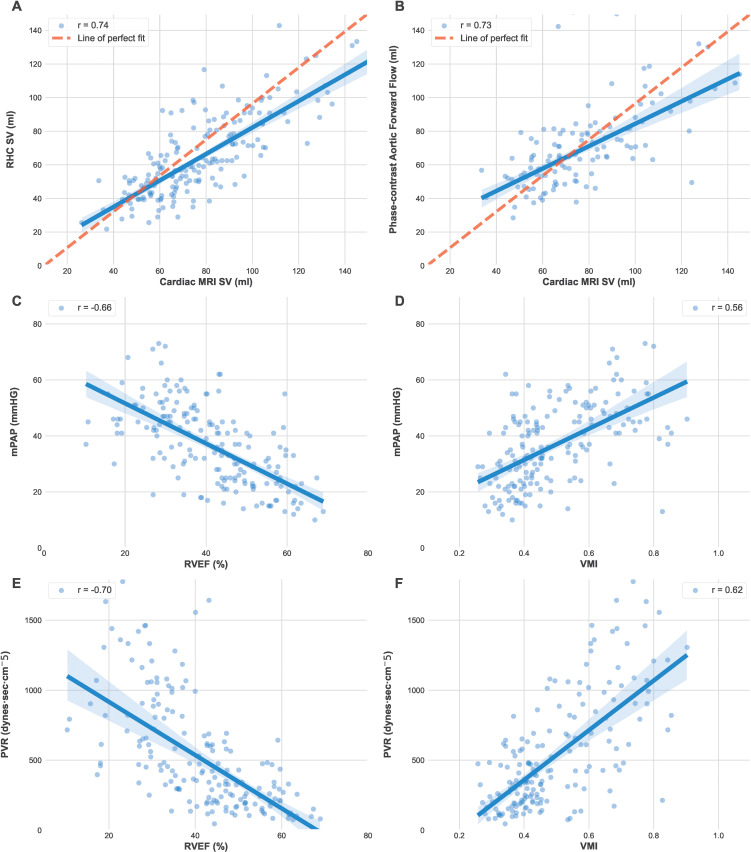
Graphs show the relationship between automatic cardiac MRI measurements, right heart catheterization (RHC) and phase-contrast aortic flow. Automatic cardiac MRI measurements were compared to **(A)** RHC stroke volume (SV) and **(B)** phase-contrast aortic flow in 178 patients of the same-day RHC cohort. **(C)** Mean pulmonary artery pressure (mPAP) was compared with right ventricle ejection fraction (RVEF) and **(D)** ventricular mass index (VMI; RV mass–to–LV mass). **(E)** Pulmonary vascular resistance (PVR) was compared to RVEF and **(F)** VMI.

The mean for cardiac MRI-estimated LV stroke volume were 78 mL ± 24 (SD) and 79 mL ± 26 for AI and manual assessments, respectively. The RHC-derived LV stroke volume was 66 mL ± 23 and the phase-contrast mean aortic forward flow volume was 68 mL ± 21. The correlation between RHC and cardiac MRI LV stroke volume (Fig 4) was higher for AI than for manual measurements (*r* = 0.74 vs 0.68, respectively; *P* = .03) (Fig 4A, Table 3). Both AI and manually derived LV stroke volume showed similar correlation with the aortic forward flow volume (*r* = 0.73 and 0.70, respectively; *P* = .29; *n* = 118), although variability is evident between the methods of stroke volume calculation, which may in part be due to technical factors and intracardiac shunts in some patients (Fig 4B). The AI-measured ventricular mass index (RV mass–to–LV mass ratio) had a higher correlation with pulmonary vascular resistance (Fig 4D) and mean pulmonary artery pressure (Fig 4F) than the manual measurements (*r* = 0.64 vs 0.44 [*P* < .001] and 0.56 vs 0.37 [*P* < .001], respectively).

**Table 3:**
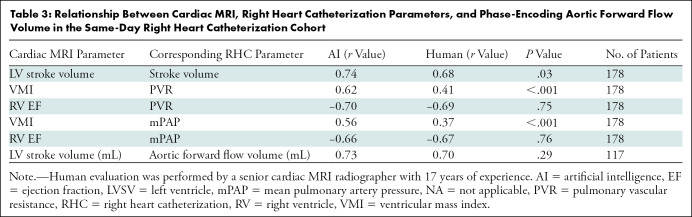
Relationship Between Cardiac MRI, Right Heart Catheterization Parameters, and Phase-Encoding Aortic Forward Flow Volume in the Same-Day Right Heart Catheterization Cohort

There were good correlations between RV ejection fraction and mean pulmonary artery pressure (Fig 4C) and pulmonary vascular resistance (Fig 4E), with no evidence of a difference between AI and manual readings. Pulmonary vascular resistance and mean pulmonary artery pressure correlated similarly with AI and manual RV ejection fraction (*P* = .75 and .76, respectively).

The correlation between AI-based cardiac MRI measurements and RHC was confirmed in 2051 patients in the ASPIRE cohort (Table E1, Fig E1 [online]).

### Mortality Prediction

Automatic cardiac MRI measurements were assessed in 3487 patients from the ASPIRE registry. The study population included patients with multiple pathologic disease, predominantly pulmonary arterial hypertension (920 of 3487; 26%), left heart disease (741 of 3487; 21%), lung diseases (480 of 3487; 13%), chronic thromboembolic pulmonary hypertension (623 of 3487; 19%), and without pulmonary hypertension (635 of 3487; 18%). During the mean follow-up period (3.8 years) 1604 of 3487 (46%) patients died. Other than RV stroke volume, all cardiac MRI parameters predicted mortality (Table 4). RV parameters including RV mass were prognostic markers in the subgroup with pulmonary arterial hypertension (*n* = 920) (Table 4). RV ejection fraction remained a significant prognostic marker in a multivariable analysis including age, World Health Organization function class, incremental shuttle walking test, RHC parameters (mean pulmonary artery pressure, pulmonary arterial wedge pressure, cardiac output, and mixed venous oxygen saturation) and cardiac MRI variables (age- and sex-corrected RV and LV ejection fraction and mass index) (Table 4). The uni- and multivariable Cox regression results for the available manual cardiac MRI measurements are provided in Table E2 (online).

**Table 4:**
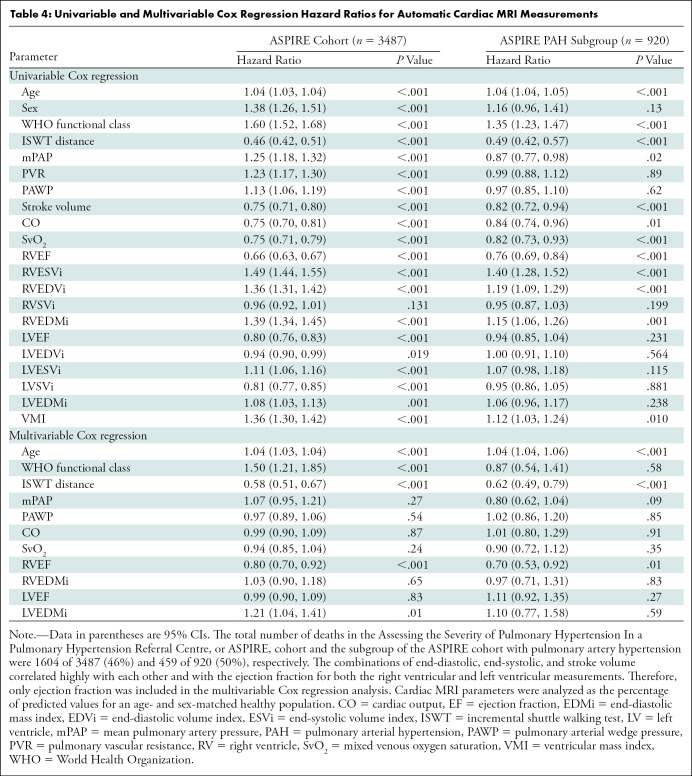
Univariable and Multivariable Cox Regression Hazard Ratios for Automatic Cardiac MRI Measurements

### Repeatability Assessment

The interstudy repeatability of cardiac MRI measurements was high for both AI and manual measurements. The automatic LV and RV volumetric and mass measurements ICC were 0.92 and 0.99, respectively. The ICC for LV and RV ejection fraction was 0.80 and 0.90, respectively (Table 5). The differences in the scan-rescan measurements were not different between AI and manual (*t* test *P* = .73 for RV ejection fraction and .8 for LV ejection fraction) (Table E3 [online]).

**Table 5:**
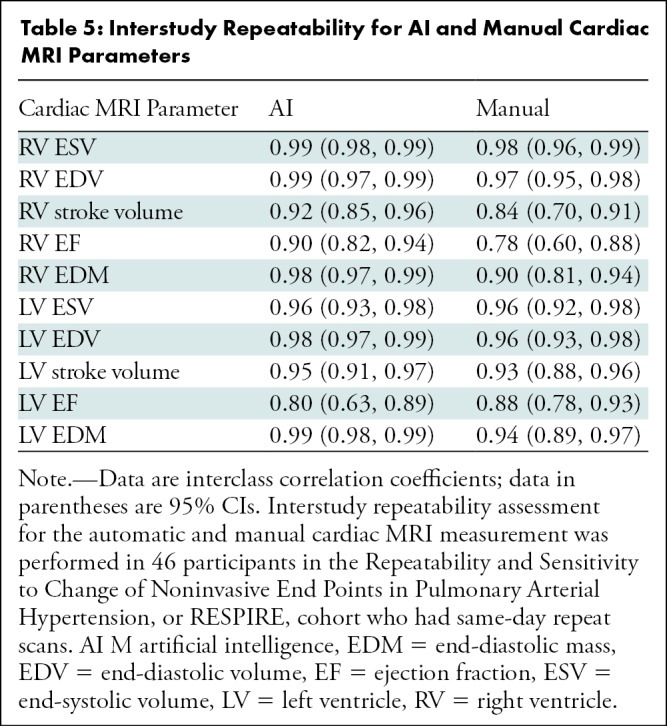
Interstudy Repeatability for AI and Manual Cardiac MRI Parameters

**Figure 5:**
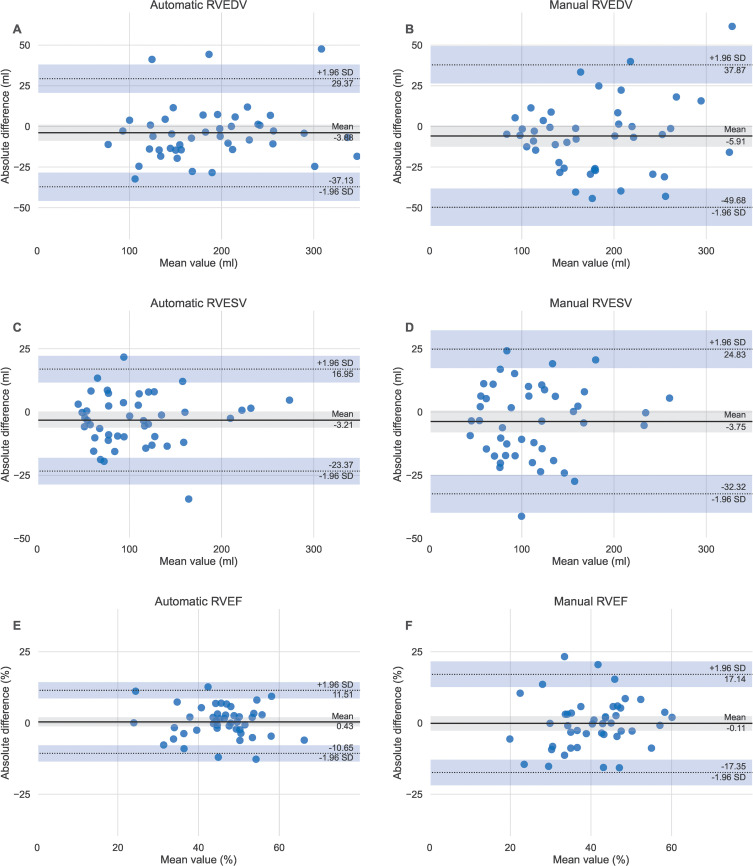
Bland-Altman plots of scan-rescan repeatability for the automatic compared to the manual right ventricular parameters. Same day scan-rescan cardiac MRIs were performed in 46 participants to compare the repeatability of the **(A, C, D)** automatic and **(B, D, F)** manual measurements. RVEDV = right ventricular end-diastolic volume, RVEF = right ventricular ejection fraction, RVESV = right ventricular end-systolic volume.

Bland-Altman plots showed strong agreement between manual and automatic measurements, with small mean absolute differences ranging between 0 mL and 4 mL in the scan-rescan measurements (Fig 5). Examples of MRI scans with higher differences are shown in Figure E2 (online).

### External Testing

There was excellent agreement between the AI and manual measurements in the multicenter and multivendor (Siemens, Philips, and GE) external data set. The interobserver ICC ranged between 0.94 and 0.99 for LV and RV volumes (Table E4 [online]). The ICC was 0.93 for LV ejection fraction and 0.94 for RV ejection fraction, and the ICCs for LV and RV mass were 0.95 and 0.92, respectively. Bland-Altman plots showed small absolute mean differences (Fig E3 [online], Fig 4).

### Segmentation Accuracy

Dice analysis showed excellent agreement in the AI and manual LV and RV epi- and endocardial end-systolic and end-diastolic contours in both the internal and external test cohorts (Table E5 [online]). The Dice values in the internal data set s ranged between 93% and 96% for the LV and 93%–95% in the RV. The Dice values were slightly lower in the external cohort and ranged from 89% to 95% in the LV and 88% to 92% in the RV.

## Discussion

Our study developed and comprehensively analyzed the performance of a fully automated biventricular cardiac MRI assessment in a large cohort of patients. We demonstrated that fully automated left ventricular (LV) stroke volume and ventricular mass index assessment had a correlation that was stronger than manual assessment with invasive hemodynamics parameters such as LV stroke volume (*r* = 0.74 vs 0.68; *P* = .03), pulmonary vascular resistance (*r* = 0.62 vs 0.41; *P* < .001), and mean pulmonary artery pressure (*r* = 0.56 vs 0.37; *P* < .001). Additionally, we showed excellent scan-rescan repeatability of artificial intelligence (AI) measurements for assessing LV and right ventricular (RV) measurements, including the more challenging RV mass (interclass correlation coefficient, 0.98; 95% CI: 0.96, 0.99). At a population level, we evaluated the prognostic value of AI-based cardiac MRI measurements and showed its ability to predict mortality in a cohort with multiple pathologic diseases, and further evaluated RV parameters in a subgroup of patients with pulmonary arterial hypertension. Finally, we have shown excellent generalizability of AI contours in an external cohort.

We included varying types and severities of conditions affecting the RV to improve the reliability of our model for automatically measuring RV function and volume. We also trained our model to recognize the RV epicardial contours to capture a variety of RV appearances, such as RV dilatation and hypertrophy, in addition to normal variations. Previous studies that assessed biventricular or focused RV short-axis segmentation used small public data sets and included no or only a limited number of patients with abnormalities of RV function (1). The largest biventricular segmentation studies were reported by Bai et al (15) and Budai et al (16) and each study included approximately 5000 participants. Bai et al (15) included healthy volunteers from the UK Biobank study, whereas Budai et al (16) included a cohort with mainly LV pathologic disease and limited RV pathologic disease because of conditions such as arrhythmogenic ventricular disease. Both studies were single center and single vendor. Our study differs in two main aspects: the AI segmentation model included large training data sets from multiple vendors (GE, Philips, and Siemens), multiple centers (Budapest and Sheffield), and multiple pathologic causes (LV and RV conditions); and automated cardiac MRI results were assessed by testing their correlation with invasive hemodynamics, prognostic ability, repeatability, and comparison to manual measurements in an external cohort. Our external test data set included patients referred to a specialist for a second opinion for complex pathologic causes.

We validated AI-derived cardiac MRI measurements against invasive hemodynamics performed on the same day. Cardiac MRI has diagnostic accuracy for pulmonary hypertension when compared to reference standard hemodynamics (17–19). The correlation between RV ejection fraction and pulmonary vascular resistance has been reported to range between −0.32 and −0.55, and the correlation with mean pulmonary artery pressure ranges between −0.28 and −0.66 (20–23). Ventricular mass index (RV mass–to–LV mass ratio) also correlates with RHC parameters ranging between 0.11 and 0.74 for pulmonary vascular resistance and from 0.53 to 0.87 for mean pulmonary artery pressure (20,22,23). Our study showed that AI-based cardiac MRI measurements correlate with RHC parameters. Particularly ventricular mass index, which is a known diagnostic and prognostic marker in pulmonary arterial hypertension, showed stronger correlation with RHC when measured automatically, indicating improved accuracy over manually measured ventricular mass index. Although some values showed a high level of disagreement, this is expected in a heterogeneous population including patients with congenital heart disease and considering the significant technical variability between the modalities compared.

The prognostic value of cardiac MRI measurements has been established in several cardiopulmonary diseases, including ischemic heart disease, cardiomyopathies, heart failure, and pulmonary arterial hypertension (24–27). In patients with pulmonary arterial hypertension, RV ejection fraction, RV end-systolic volume index, and RV end-diastolic volume index were shown to predict mortality and clinical worsening in a meta-analysis of almost 2000 patients (28). Our study confirmed the prognostic ability of automatic cardiac MRI measurements in a large cohort of 3417 patients with multiple pathologic diseases, including 920 patients with pulmonary arterial hypertension. RV ejection fraction, RV end-systolic volume index, RV end-diastolic volume index and RV end-diastolic mass index predicted death in pulmonary arterial hypertension when corrected for age and sex. RV end-diastolic mass index is not assessed in commercial software packages but can provide useful prognostic information, particularly in pulmonary hypertension. Additionally, we showed that automatically measured RV ejection fraction is a statistically significant prognostic marker in pulmonary arterial hypertension when added to functional assessment (World Health Organization functional class and walking test) and right heart catheterization parameters.

Although our analysis showed that differences between the automatic and manual measurements were not statistically significant, these differences can be relatively large (eg, 6% difference in ejection fraction). Therefore, we believe that establishing normal ranges of AI segmentation is important. Additionally, despite similar repeatability between the automatic and manual segmentation, consistent differences were noted in the manual segmentation of the scan-rescan cohort, such as excluding portions of the right ventricular outflow tract. Whereas this consistency maintained excellent repeatability, the manual segmentation was less accurate. The scan-rescan segmentation was performed by a cardiac MRI practitioner not involved in the AI model training, highlighting the existence of subjective differences in the interpretation of the base of the heart even within the same institution. Furthermore, the AI segmentation fails in some patients, showing the need for further training. Failed AI segmentation will be continuously identified and incorporated in future training rounds to improve the accuracy of the model.

Our study had limitations. First, the validation, including comparison with heart catheterization and prediction of mortality, was performed in a single center with two MRI systems and limited cohort description. Second, direct comparison between AI and manual measurements in the large ASPIRE cohort for RHC correlation and mortality prediction could not be performed because of differences in handling trabeculations. Third, the segmentation algorithm cannot be made publicly available because the deep learning code would require extensive documentation and compatibility scripts to enable the application by external parties. However, we encourage readers to contact the corresponding author for research access to the Mass software and the AI segmentation tool.

In conclusion, we described a human-in-the-loop artificial intelligence (AI) approach to develop a biventricular cardiac MRI assessment tool. We provided a comprehensive evaluation of AI-based cardiac MRI measurements in a large cohort of patients with a wide spectrum of right and left ventricular pathologic abnormalities and normal variants. Fully automatic cardiac MRI assessment correlates with invasive hemodynamics and has prognostic value. Training to target apex errors and more extreme pathologic abnormalities could advance the AI method further. Future research that uses cardiac MRI as a clinical end point can benefit from the high repeatability and generalizability of AI measurements.

## References

[1] Chen C, Qin C, Qiu H, et al. Deep Learning for Cardiac Image Segmentation: A Review. Front Cardiovasc Med 2020;7:25. 32195270 10.3389/fcvm.2020.00025 PMC7066212

[2] Tao Q, Yan W, Wang Y, et al. Deep Learning-based Method for Fully Automatic Quantification of Left Ventricle Function from Cine MR Images: A Multivendor, Multicenter Study. Radiology 2019;290(1):81–88. 30299231 10.1148/radiol.2018180513

[3] Petitjean C, Zuluaga MA, Bai W, et al. Right ventricle segmentation from cardiac MRI: a collation study. Med Image Anal 2015;19(1):187–202. 25461337 10.1016/j.media.2014.10.004

[4] Kiely DG, Elliot CA, Sabroe I, Condliffe R. Pulmonary hypertension: diagnosis and management. BMJ 2013;346:f2028. 23592451 10.1136/bmj.f2028

[5] Hurdman J, Condliffe R, Elliot CA, et al. ASPIRE registry: assessing the Spectrum of Pulmonary hypertension Identified at a REferral centre. Eur Respir J 2012;39(4):945–955. 21885399 10.1183/09031936.00078411

[6] Swift AJ, Wilson F, Cogliano M, et al. Repeatability and sensitivity to change of non-invasive end points in PAH: the RESPIRE study. Thorax 2021;76(10):1032–1035. 33632769 10.1136/thoraxjnl-2020-216078 PMC8461450

[7] Mongan J, Moy L, Kahn CE Jr. Checklist for Artificial Intelligence in Medical Imaging (CLAIM): A Guide for Authors and Reviewers. Radiol Artif Intell 2020;2(2):e200029. 33937821 10.1148/ryai.2020200029 PMC8017414

[8] Abadi M, Agarwal A, Barham P, et al. TensorFlow: Large-scale machine learning on heterogeneous distributed systems. arXiv preprint arXiv:1603.04467. http://arxiv.org/abs/1603.04467. Posted 2016. Accessed May 23, 2022.

[9] Maceira AM, Prasad SK, Khan M, Pennell DJ. Normalized left ventricular systolic and diastolic function by steady state free precession cardiovascular magnetic resonance. J Cardiovasc Magn Reson 2006;8(3):417–426. 16755827 10.1080/10976640600572889

[10] Maceira AM, Prasad SK, Khan M, Pennell DJ. Reference right ventricular systolic and diastolic function normalized to age, gender and body surface area from steady-state free precession cardiovascular magnetic resonance. Eur Heart J 2006;27(23):2879–2888. 17088316 10.1093/eurheartj/ehl336

[11] Steiger JH. Tests for comparing elements of a correlation matrix. Psychol Bull 1980;87(2):245–251.

[12] Vallat R. Pingouin: statistics in Python. J Open Source Softw 2018;3(31):1026.

[13] Davidson-Pilon C, Kalderstam J, Jacobson N, et al. CamDavidsonPilon/lifelines: v0.25.9. Published February 5, 2021. Accessed May 23, 2022.

[14] Hunter JD. Matplotlib: A 2D Graphics Environment. Comput Sci Eng 2007;9(3):90–95.

[15] Bai W, Sinclair M, Tarroni G, et al. Automated cardiovascular magnetic resonance image analysis with fully convolutional networks. J Cardiovasc Magn Reson 2018;20(1):65. 30217194 10.1186/s12968-018-0471-x PMC6138894

[16] Budai A, Suhai FI, Csorba K, et al. Fully automatic segmentation of right and left ventricle on short-axis cardiac MRI images. Comput Med Imaging Graph 2020;85:101786. 32866695 10.1016/j.compmedimag.2020.101786

[17] Chen H, Xiang B, Zeng J, Luo H, Yang Q. The feasibility in estimating pulmonary vascular resistance by cardiovascular magnetic resonance in pulmonary hypertension: A systematic review and meta-analysis. Eur J Radiol 2019;114:137–145. 31005164 10.1016/j.ejrad.2019.03.014

[18] Rajaram S, Swift AJ, Capener D, et al. Comparison of the diagnostic utility of cardiac magnetic resonance imaging, computed tomography, and echocardiography in assessment of suspected pulmonary arterial hypertension in patients with connective tissue disease. J Rheumatol 2012;39(6):1265–1274. 22589263 10.3899/jrheum.110987

[19] Ullah W, Minalyan A, Saleem S, et al. Comparative accuracy of non-invasive imaging versus right heart catheterization for the diagnosis of pulmonary hypertension: A systematic review and meta-analysis. Int J Cardiol Heart Vasc 2020;29:100568. 32642551 10.1016/j.ijcha.2020.100568 PMC7334462

[20] Swift AJ, Rajaram S, Condliffe R, et al. Diagnostic accuracy of cardiovascular magnetic resonance imaging of right ventricular morphology and function in the assessment of suspected pulmonary hypertension results from the ASPIRE registry. J Cardiovasc Magn Reson 2012;14(1):40. 22720870 10.1186/1532-429X-14-40 PMC3419131

[21] Alunni JP, Degano B, Arnaud C, et al. Cardiac MRI in pulmonary artery hypertension: correlations between morphological and functional parameters and invasive measurements. Eur Radiol 2010;20(5):1149–1159. 20094890 10.1007/s00330-009-1664-3

[22] Zhang Z, Wang M, Yang Z, et al. Noninvasive prediction of pulmonary artery pressure and vascular resistance by using cardiac magnetic resonance indices. Int J Cardiol 2017;227:915–922. 27913006 10.1016/j.ijcard.2016.10.068

[23] Ali ER, Mohamad AM. Diagnostic accuracy of cardiovascular magnetic resonance imaging for assessment of right ventricular morphology and function in pulmonary artery hypertension. Egypt J Chest Dis Tuberc 2017;66(3):477–486.

[24] Klem I, Shah DJ, White RD, et al. Prognostic value of routine cardiac magnetic resonance assessment of left ventricular ejection fraction and myocardial damage: an international, multicenter study. Circ Cardiovasc Imaging 2011;4(6):610–619. 21911738 10.1161/CIRCIMAGING.111.964965

[25] Mordi I, Bezerra H, Carrick D, Tzemos N. The Combined Incremental Prognostic Value of LVEF, Late Gadolinium Enhancement, and Global Circumferential Strain Assessed by CMR. JACC Cardiovasc Imaging 2015;8(5):540–549. 25890580 10.1016/j.jcmg.2015.02.005

[26] Rodriguez-Palomares JF, Gavara J, Ferreira-González I, et al. Prognostic Value of Initial Left Ventricular Remodeling in Patients With Reperfused STEMI. JACC Cardiovasc Imaging 2019;12(12):2445–2456. 31202752 10.1016/j.jcmg.2019.02.025

[27] Swift AJ, Capener D, Johns C, et al. Magnetic Resonance Imaging in the Prognostic Evaluation of Patients with Pulmonary Arterial Hypertension. Am J Respir Crit Care Med 2017;196(2):228–239. 28328237 10.1164/rccm.201611-2365OC PMC5519970

[28] Alabed S, Shahin Y, Garg P, et al. Cardiac-MRI Predicts Clinical Worsening and Mortality in Pulmonary Arterial Hypertension: A Systematic Review and Meta-Analysis. JACC Cardiovasc Imaging 2021;14(5):931–942 [Published correction appears in JACC Cardiovasc Imaging 2021;14(4):884.]. 33008758 10.1016/j.jcmg.2020.08.013 PMC7525356

